# Tapping into the endocannabinoid system to ameliorate acute inflammatory flares and associated pain in mouse knee joints

**DOI:** 10.1186/s13075-014-0437-9

**Published:** 2014-09-27

**Authors:** Eugene Krustev, Allison Reid, Jason J McDougall

**Affiliations:** Departments of Pharmacology and Anaesthesia, Pain Management and Perioperative Medicine, Dalhousie University, 5850 College Street, Halifax, NS B3H 4R2 Canada

## Abstract

**Introduction:**

During the progression of rheumatoid arthritis (RA), there are frequent but intermittent flares in which the joint becomes acutely inflamed and painful. Although a number of drug therapies are currently used to treat RA, their effectiveness is variable and side effects are common. Endocannabinoids have the potential to ameliorate joint pain and inflammation, but these beneficial effects are limited by their rapid degradation. One enzyme responsible for endocannabinoid breakdown is fatty acid amide hydrolase (FAAH). The present study examined whether URB597, a potent and selective FAAH inhibitor, could alter inflammation and pain in a mouse model of acute synovitis.

**Methods:**

Acute joint inflammation was induced in male C57BL/6 mice by intra-articular injection of 2% kaolin/2% carrageenan. After 24 hr, articular leukocyte kinetics and blood flow were used as measures of inflammation, while hindlimb weight bearing and von Frey hair algesiometry were used as measures of joint pain. The effects of local URB597 administration were then determined in the presence or absence of either the cannabinoid (CB)1 receptor antagonist AM251, or the CB2 receptor antagonist AM630.

**Results:**

URB597 decreased leukocyte rolling and adhesion, as well as inflammation-induced hyperaemia. However, these effects were only apparent at low doses and the effects of URB597 were absent at higher doses. In addition to the anti-inflammatory effects of URB597, fatty acid amide hydrolase (FAAH) inhibition improved both hindlimb weight bearing and von Frey hair withdrawal thresholds. The anti-inflammatory effects of URB597 on leukocyte rolling and vascular perfusion were blocked by both CB1 and CB2 antagonism, while the effect on leukocyte adherence was independent of cannabinoid receptor activation. The analgesic effects of URB597 were CB1 mediated.

**Conclusions:**

These results suggest that the endocannabinoid system of the joint can be harnessed to decrease acute inflammatory reactions and the concomitant pain associated with these episodes.

## Introduction

Rheumatoid arthritis (RA) is an inflammatory disorder that is one of the leading causes of disability worldwide [1]. Although the pathological presentation of RA can vary between patients, a prominent characteristic of the disease is the occurrence of acute inflammatory flares with concomitant pain. During inflammatory flares, blood flow is increased to the inflamed area and leukocytes are recruited to the affected joint. These events can lead to a potentiation of the inflammatory response; therefore, pharmacotherapeutics that decrease both the synovitis and joint pain would be extremely beneficial for the clinical management of RA.

During synovitis, pro-inflammatory molecules released into the joint initiate local inflammatory vasodilatation and increased vascular permeability [2]. The migration of immune cells into inflamed tissues involves a multi-step process, which requires biochemical interactions between the leukocytes and the local microvasculature. During inflammation, vascular endothelial cells begin to express cell adhesion molecules (CAMs) that bind other CAMs expressed on the surface of passing leukocytes. These interactions initiate the capture of activated leukocytes, which commence a rolling behaviour where the cells move slower than the surrounding circulation. As leukocyte velocity continues to decrease, these cells eventually stop and adhere to the intravascular wall. Finally, adherent leukocytes are able to exit the blood vessels and enter the surrounding tissue, where they can release various mediators that influence local inflammation [3].

The number one concern of RA patients is safe and effective alleviation of the chronic pain that accompanies the disease. During acute synovitis, algogenic mediators are released from extravasated immune cells and primary afferent nerve terminals. The accumulation of these pain-causing agents within the joint leads to the sensitization of mechanosensory nerves and the awakening of silent nociceptors, such that even normal joint movements become painful [4,5]. In the rat knee joint, inflammatory neuropeptides that have been shown to induce peripheral sensitization and cause pain include substance P [6], vasoactive intestinal peptide [7], nociceptin [8] and pituitary adenylate cyclase-activating polypeptide [9]. Conversely, very little is known regarding the activity of endogenous analgesic mediators in joint tissues. The endogenous opioid peptide endomorphin-1 was found to reduce peripheral sensitization in acutely inflamed rat knees [10], as well as ameliorating joint inflammation [11]. The serine proteinase cathepsin G has also been shown to reduce nociception in normal rat knees [12].

Cannabinoids are a family of molecules related to the biologically active components of *Cannabis sativa*, and have the potential to alleviate both inflammation and pain [13-15]. Cannabinoids are classified by their ability to activate cannabinoid type 1 (CB1) [16] and/or type 2 (CB2) receptors. The cannabinoid system is a promising target for treating arthritis because of the localization of CB1 receptors on joint primary afferents [17] and CB2 receptors in the synovium [18]. In a rat model of osteoarthritis (OA), articular CB1 receptor activation significantly decreased the nociceptor firing rate [19], while the CB2 receptor agonist GW405833 unexpectedly sensitized joint afferents [18], although off-target drug effects may have produced this latter response. Local administration of cannabinoids has also been found to increase synovial blood flow [20], indicating that the synovial cannabinoid system can increase joint inflammation. Although cannabinoids were first discovered in plants (phytocannabinoids) [13], recent research has uncovered several endogenous cannabinoid molecules, the most well studied being N-arachidonoylethanolamine (anandamide) [14,21]. Anandamide is known to be both anti-inflammatory [14] and analgesic [15]; however, these beneficial effects are limited, due to the rapid metabolism of anandamide by enzymes like fatty acid amide hydrolase (FAAH) [22]. In addition to anandamide, FAAH is also responsible for the metabolism of other fatty acid amides like N-oleoylethanolamine (OEA) and N-palmitoylethanolamine (PEA) [23]. Inhibiting FAAH increases tissue anandamide levels *in vivo* [24], and FAAH inhibitors are capable of decreasing pain in rodent models of OA [25].

The current study aimed to test the effects of local FAAH inhibition, using the potent and selective FAAH inhibitor URB597, on blood flow, leukocyte trafficking and pain in a mouse model of acute arthritic flares.

## Methods

### Animals

Male C57BL/6 mice (n =175; 21 to 32 g; six to eight weeks old; Charles River Laboratories Inc., Senneville, QC, Canada) were housed at 22 ± 2°C on a 12:12 hr light:dark cycle (light-on from 7:00 to 19:00). Cages were lined with woodchip bedding and animals were provided with environmental enrichment. Standard lab chow and water were provided *ad libitum*. The experimental protocols were approved by the Dalhousie University Committee on Laboratory Animals, which acts in accordance with the standards put forth by the Canadian Council for Animal Care.

### Kaolin-carrageenan inflammation

All animals had an acute unilateral inflammation induced by injection of kaolin and carrageenan into the right knee joint. Animals were deeply anaesthetized (2 to 4% isoflurane; 100% oxygen at 1 L/min) and an acceptable plane of anaesthesia was confirmed by failure to produce a hindpaw withdrawal reflex. The right knee was shaved and the knee joint diameter was measured using a digital micrometer (Mituyoto Instruments, Tokyo, Japan). The knee area was then swabbed with 100% ethanol and 10 μl of 2% kaolin was injected into the intra-articular space. The knee was then manually extended and flexed for 10 min to disperse the kaolin throughout the joint and irritate the synovium. Next, 10 μl of 2% carrageenan was injected in the same manner and was followed by 30 s of hindlimb flexion and extension. Animals were returned to their cages for 24 hr. Knee joint diameter was then reassessed to confirm positive inflammation.

### Surgical preparation for vascular assessment

The following preparation was conducted before intravital microscopy (IVM) and laser speckle contrast analysis (LASCA) of the inflamed knee. Animals were anaesthetized by an intraperitoneal injection of urethane (25% in saline; 0.3 to 0.4 ml) and a surgical plane of anaesthesia was confirmed by failure to elicit a hindpaw withdrawal reflex. A longitudinal incision was made in the skin of the neck to expose the trachea, left carotid artery and left jugular vein, which were individually cannulated. PE-60 tubing was used for the tracheal cannulation while PE-10 tubing, filled with heparinized saline (1U/ml), was used for the blood vessel cannulations. The capsular microcirculation of the right knee was exposed by surgically removing a small ellipse of overlying skin (<1 cm long; <0.5 cm wide), and the knee was immobilized. Physiological buffer (37°C) was immediately and continuously perfused over the exposed joint.

### Intravital microscopy

IVM was used to assess leukocyte trafficking within the microcirculation of the knee joint, as previously described [26]. After surgical preparation, the synovial microcirculation was visualized under incident fluorescent light using a Leica DM2500 microscope with a HCX APO L 20X objective and a HC Plan 10X eyepiece (Leica Microsystems Inc., Richmond Hill ON, Canada; final magnification 200X). *In vivo* leukocyte staining was achieved by intravenous administration of 0.05% rhodamine 6G (0.05 ml). Straight, unbranched, postcapillary venules (15 to 50 μm in diameter) overlying the knee capsule were selected for visualization. Videos (1 min duration) of leukocyte kinetics were captured using a BC-71 AVT camera (Horn Imaging, Aalen, Germany).

Two measures of leukocyte-endothelial interactions were used to assess inflammation: (i) number of rolling leukocytes and (ii) number of adherent leukocytes per 50 μm of vessel. Rolling leukocytes were defined as rhodamine 6G-stained cells travelling slower than the surrounding flow of blood in the vessel of interest. The rolling leukocyte measure was obtained by counting the number of rolling leukocytes per minute to pass an arbitrary line perpendicular to the vessel of interest. Adherent leukocytes were defined as rhodamine 6G-stained cells that remained stationary for a minimum of 30 s. Total leukocyte adhesion was quantified by counting the number of adherent cells within a 50 μm portion of the vessel. A baseline measure was first taken to observe leukocyte kinetics before drug administration. Following this measure, either vehicle, URB597 (0.3 to 30 mg/kg), URB597 (0.3 mg/kg) + AM251 (CB1 antagonist, 0.2 mg/kg) or URB597 (0.3 mg/kg) + AM630 (CB2 antagonist, 0.2 mg/kg) was applied directly over the exposed knee joint. Doses were chosen based on effective dosing regimens as previously reported [18-20,25]. For all URB597 + antagonist trials in this study, the antagonists were administered 10 min before URB597. Antagonist alone control trials were also conducted. Subsequent recordings were taken 5, 10, 20, 30 and 60 min after URB597 administration.

### Laser speckle contrast analysis

In order to quantify microvascular perfusion, knee joint blood flow was assessed using a PeriCam PSI System (Perimed Inc., Ardmore, PA, USA). Recordings were taken at a working distance of 10 cm with a frame rate of 25 images per second, which were then averaged to create one perfusion image per second. Following a baseline recording of 1 min, a 100 μl bolus of URB597 (0.3 to 30 mg/kg) was applied directly to the knee joint, with each dose being applied in separate cohorts of animals. The effect of AM251 or AM630 (0.2 mg/kg) on URB597 perfusion responses was also tested in different groups of mice. Mean perfusion (1 min sampling time) was measured at 5, 10, 20, 30 and 60 min following drug treatment. A dead scan of the knee was taken *post mortem* and this value was subtracted from all measurements to account for any optical noise in the tissue.

### Hindlimb incapacitance

Mice were habituated to the hindlimb incapacitance testing apparatus (Linton Instrumentation, Diss, UK) for three consecutive days prior to baseline measurement. Weight distribution measurements of one-second duration were taken while the animal was standing on both hindlimbs, with one hindpaw on each of the two force plates. Baseline weight-bearing measurements were taken before and 24 hr after induction of inflammation. Separate cohorts of mice were then treated with either vehicle, URB597 (3 mg/kg), URB597 (3 mg/kg) + AM251 (0.2 mg/kg), or URB597 (3 mg/kg) + AM630 (0.2 mg/kg), applied subcutaneously (s.c.) over the inflamed knee joint, or URB597 (3 mg/kg) applied s.c. over the contralateral knee joint. Test measurements (average of six measures per time point) were then taken at 30, 60, 90 and 180 min following drug administration.

### von Frey hair mechanosensitivity

The same animals underwent concurrent secondary allodynia testing as a behavioural measure of referred pain. Ipsilateral hindpaw mechanosensitivity was assessed by plantar application of von Frey hair filaments using a modification of the Dixon up-and-down method [27]. Animals were placed in elevated Plexiglas chambers on metal mesh flooring allowing access to the paws. After allowing the animal to acclimate until exploratory behaviour ceased, a von Frey hair was applied perpendicular to the plantar surface of the hindpaw (avoiding the toe pads) until the hair just bent, and the hair was held in place for three seconds. If there was a positive response (that is withdrawal, shake or lick of the hindpaw), the next lower strength hair was applied; if there was no response, the next higher strength hair was applied up to a maximum cutoff level, which corresponded to a 4 g bending force. After the first difference in response was observed, four more measurements were made and the pattern of responses were converted to a 50% withdrawal threshold calculated using the following formula: 10^[*Xf*+*k*δ]^/10,000; where *Xf* = value (in log units) of the final von Frey hair used, *k* = tabular value for the pattern of the last six positive/negative responses, and δ = mean difference (in log units) between stimuli. A time course at 30, 60, 90 and 180 min following drug administration was constructed.

### Drugs and reagents

URB597 (FAAH inhibitor; [3-(3-carbamoylphenyl)phenyl] N-cyclohexylcarbamate), AM251 (CB1 receptor antagonist; 1-(2,4-dichlorophenyl)-5-(4-iodophenyl)-4-methyl-N-piperidin-1-ylpyrazole-3-carboxamide) and AM630 (CB2 receptor antagonist; 6-iodo-2-methyl-1-(2-morpholin-4-ylethyl)indol-3-yl]-(4-methoxyphenyl)methanone) were obtained from Cayman Chemicals (Ann Arbor, MI, USA). Rhodamine 6G, cremophor, dimethyl sulphoxide (DMSO), urethane, carrageenan and kaolin were obtained from Sigma-Aldrich (St. Louis, MO, USA). Solutions of URB597 (0.3 to 30 mg/kg), AM251 (0.2 mg/kg) and AM630 (0.2 mg/kg) were prepared in vehicle (1:1:8; DMSO:cremophor:saline) on the day of use. Rhodamine 6G (0.05%) was dissolved in saline. Physiological buffer (135 mM NaCl, 20 mM NaHCO3, 5 mM KCl, 1 mM MgSO4*7H2O, pH =7.4) was prepared in house. Solutions of carrageenan (2%) and kaolin (2%) were dissolved in saline and stored at 4°C.

### Statistical analysis

Data conformed to a Gaussian distribution (Kolmogrov-Smirnov normality test) and were, therefore, tested with parametric statistics. For the IVM and LASCA experiments, time-course data were analyzed using a two-way analysis of variance (ANOVA), with time and drug treatment as the variables. The time at which URB597 had the greatest effect, when compared to vehicle, was selected and further analysis focused on this time point. For the dose-response profiles, groups of data were compared against vehicle using a one-way ANOVA with a Dunnett’s *post hoc* test. All other analysis of drug effects were compared to URB597 using a one-way ANOVA with a Bonferroni correction. Knee diameter data were analyzed by a paired two-tailed Student’s *t* test. A *P* value <0.05 was considered statistically significant. Data are expressed as means ± standard error of the mean (SEM).

## Results

### Knee diameter

To ensure adequate inflammation, knee joint diameter was measured before and 24 hr after kaolin/carrageenan injection. Kaolin/carrageenan inflammation resulted in an average of 0.60 ± 0.02 mm (15.5%) increase in knee diameter 24 hr post injection (3.71 ± 0.01 mm before; 4.31 ± 0.02 mm after; *P* <0.0001; *n =*175).

### Mean arterial pressure

During IVM and LASCA measurements, mean arterial pressure did not change in response to drug administration and was found to be comparable between all experimental groups.

### Leukocyte trafficking

**Figure 1:**
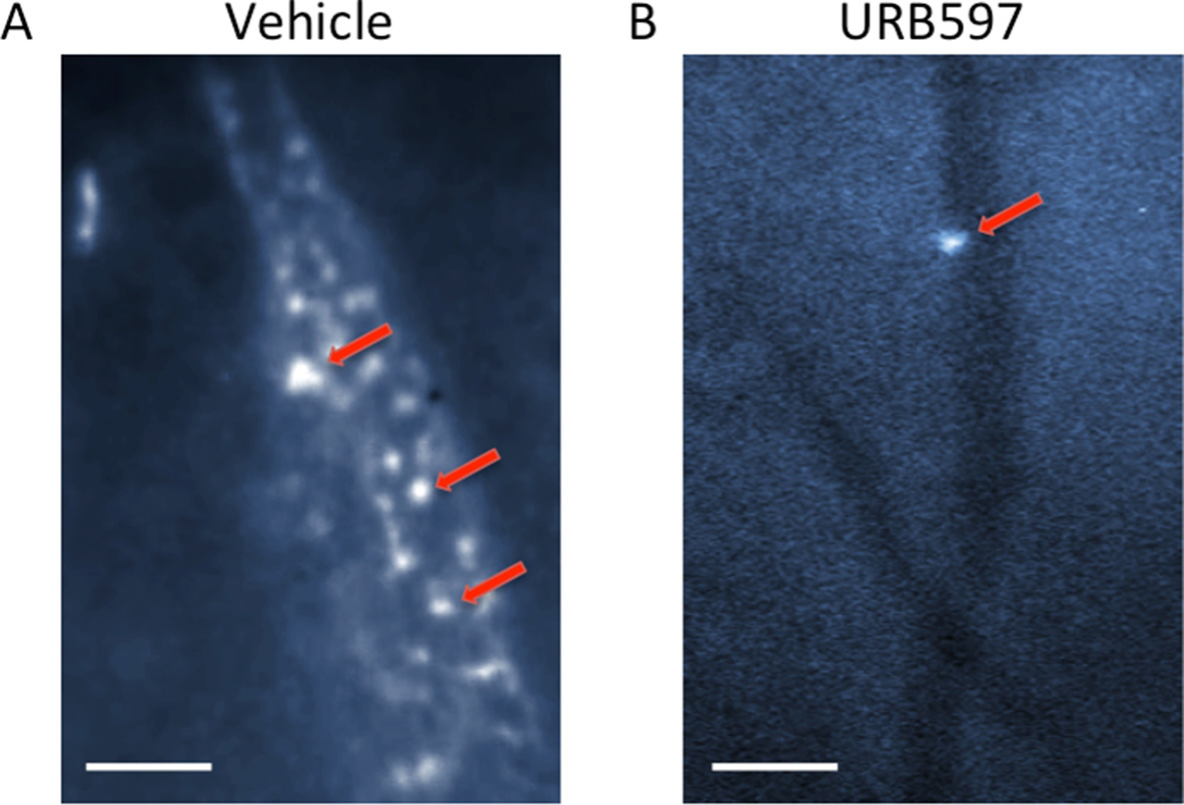
**Intravital microscopy images showing circulating leukocytes in the anteromedial aspect of acutely inflamed mouse knees in response to either (A) vehicle or (B) the FAAH inhibitor URB597.** Arrows indicate stained leukocytes. Scale bar =50 μm. FAAH, fatty acid amide hydrolase.

**Figure 2:**
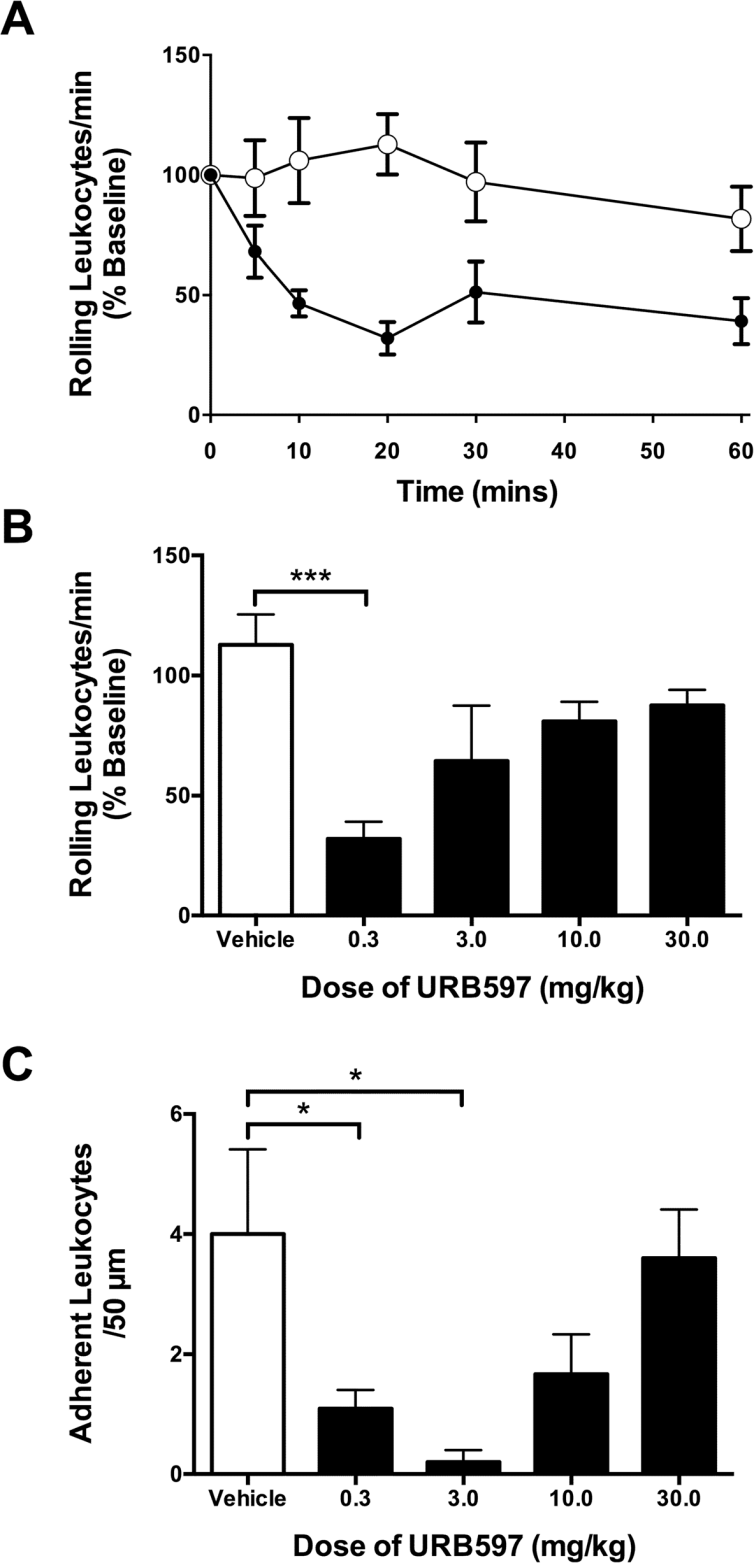
**The effects of URB597 on leukocyte trafficking in the inflamed knee. (A)** Time course of the effect of locally administered URB597 (closed circles) vs. vehicle (open circles) on leukocyte rolling. There was a significant reduction in the number of rolling leukocytes after URB597 administration, when compared to vehicle (*P* <0.0001; two-way ANOVA; *n* =7 to 11). **(B)** Dose-response profile of the effect of URB597 on leukocyte rolling 20 min after administration. URB597 (0.3 mg/kg) significantly decreased leukocyte rolling, when compared to vehicle, but this effect was diminished at higher doses. **(C)** Dose-response profile for the effects of URB597 on leukocyte adhesion 20mins after administration. URB597 (0.3 and 3.0 mg/kg) significantly inhibited leukocyte adhesion, when compared to vehicle; however, this effect was attenuated at higher doses. Data are means ± SEM. ^*^ *P* <0.05, ^**^ *P* <0.01, ^***^ *P* <0.001 one-way ANOVA with Dunnett’s *post hoc* test vs. vehicle; *n* =5 to 11. ANOVA, analysis of variance; SEM, standard error of the mean.

**Figure 3:**
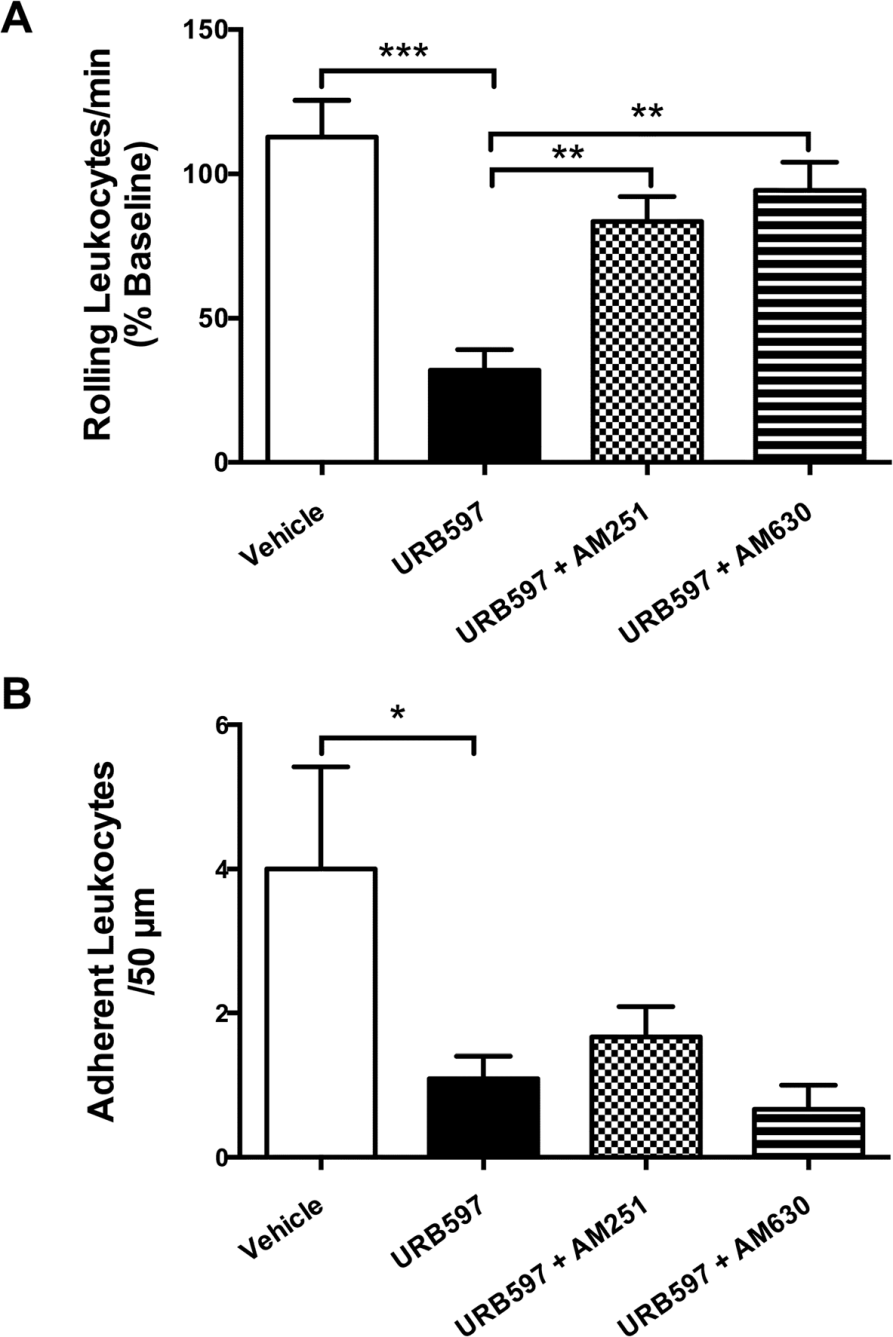
**The contribution of cannabinoid receptors to the effects of URB597 on leukocyte trafficking in the inflamed knee. (A)** Number of rolling leukocytes per minute 20 min after administration of URB597 (0.3 mg/kg) to acutely inflamed knees. Local application of the drug significantly decreased the number of rolling leukocytes and this effect was blocked by local pretreatment with the CB1 receptor antagonist AM251 (0.2 mg/kg) and the CB2 receptor antagonist AM630 (0.2 mg/kg). **(B)** Number of adherent leukocytes per 50 μm of vessel 20 min after URB597 administration. Leukocyte adhesion was significantly reduced by URB597 administration and this effect was not blocked by either cannabinoid receptor antagonist. Data are means ± SEM. ^*^ *P* <0.05, one-way ANOVA with Bonferroni’s *post hoc* test vs. URB597 (0.3 mg/kg); *n* =6 to 11. ANOVA, analysis of variance; SEM, standard error of the mean.

Over the course of one hour, URB597 (0.3 mg/kg) significantly decreased leukocyte rolling when compared to vehicle (*P* <0.0001; *n* =7 to 11; Figures 1 and 2A). The most robust effect was observed 20 min after administration; therefore all subsequent measurements focused on this time point. Low-dose URB597 (0.3 mg/kg) caused an average of 68.0 ± 6.8% (*n =*11) decrease in leukocyte rolling 20 min after administration (Figure 2B). This inhibition of rolling was significantly different from vehicle-treated animals (*P* <0.001; *n =*7 to 11; Figure 2B) and was blocked by both AM251 and AM630 (*P* <0.01; *n =*6; Figure 3A). Interestingly, higher doses of URB597 (3 to 30 mg/kg) had no inhibitory effect on leukocyte rolling (Figure 2B).

At 20 min, URB597 (0.3 and 3.0 mg/kg) also significantly decreased the number of adherent leukocytes compared to vehicle (*P* <0.05; *n =*6; Figure 2C). Higher doses of URB597 (10 to 30 mg/kg), however, were less effective (*n* =5 to 6; Figure 2C). The inhibitory effect of URB597 (0.3 mg/kg) on leukocyte adhesion was not blocked by AM251 (*P* >0.05; *n =*6; Figure 3B) nor AM630 pretreatment (*P* >0.05; *n* =6; Figure 3B).

When administered alone, neither antagonist had any effect on leukocyte rolling or adherence, when compared to vehicle (*P* >0.05; *n* =6 to 7; data not shown).

### Microvascular perfusion

**Figure 4:**
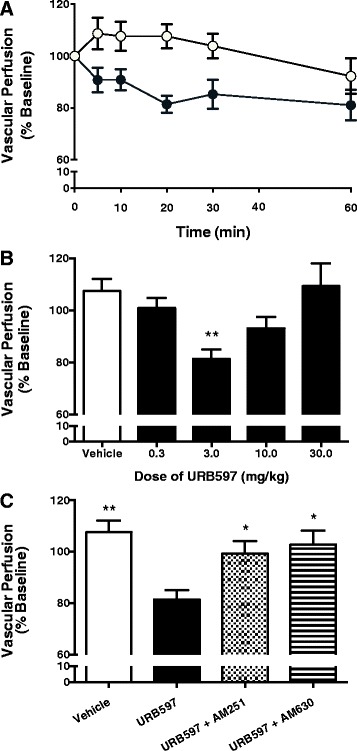
**Laser speckle contrast analysis of the inflamed mouse knee joint. (A)** Time course of the effect of locally administered URB597 (closed circles) vs. vehicle (open circles) on vascular perfusion. There was a significant reduction in local blood flow after URB597 administration, when compared to vehicle. *P* <0.0001; two-way ANOVA; *n* =6 to 7. **(B)** Dose-response profile for the effects of URB597 20 mins after administration. Local blood flow was significantly reduced by only low-dose URB597 (3.0 mg/kg). ^**^ *P* <0.01 one-way ANOVA with Dunnett’s *post hoc* test vs. vehicle; *n* =5 to 7. **(C)** Both AM251 (0.2 mg/kg) and AM630 (0.2 mg/kg) blocked the effect of URB597 (3.0 mg/kg) on blood flow in the inflamed mouse knee joint. Data are means ± SEM.^*^ *P* <0.05, one-way ANOVA with Bonferroni’s *post hoc* test vs. URB597 3.0 mg/kg; n =6 to 8. ANOVA, analysis of variance; SEM, standard error of the mean.

Local administration of URB597 (3.0 mg/kg) decreased microvascular perfusion, when compared to vehicle treatment (*P* <0.01; *n* =6 to 10; Figure 4A). This effect was most prominent at 20 min, where URB397 (3.0 mg/kg) decreased microvascular perfusion by an average of 19.6 ± 3.6% (*n* =7; Figure 4B). The inhibitory effect of URB597 on joint hyperaemia was blocked by both AM251 and AM630 (*P* <0.05; *n* =6 to 10; Figure 4C). High-dose URB597 (10 to 30 mg/kg) was ineffective at reducing inflamed joint hyperaemia (*n* =5 to 7; Figure 4B).

### Hindlimb weight bearing

**Figure 5:**
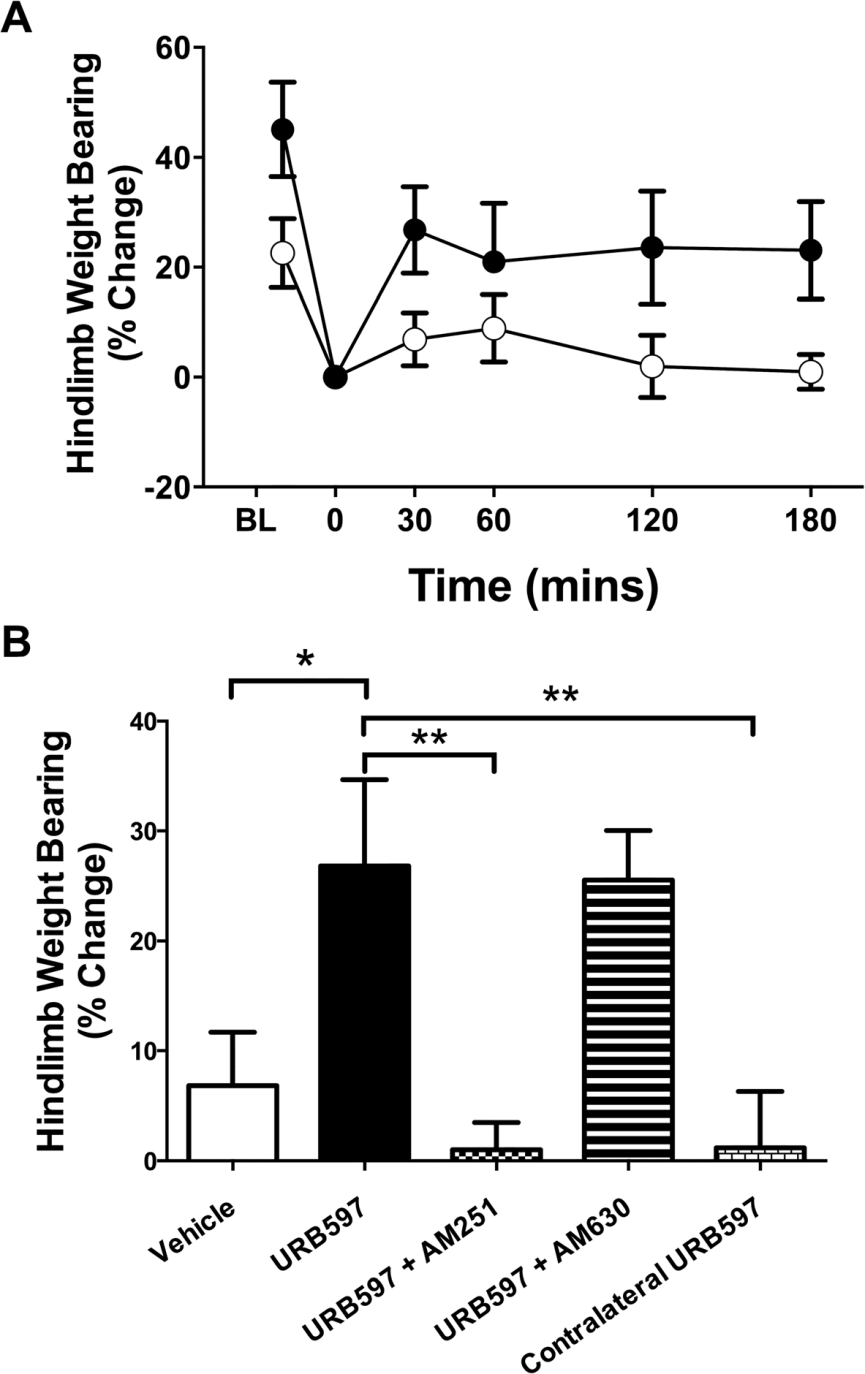
**Hindlimb weight bearing in mice with acute experimental joint inflammation. (A)** Time course of the effects of URB597 (3.0 mg/kg) on inflamed hindlimb weight bearing (baseline (BL) values represent pre-inflammation induction). Over the course of 3 hr, URB597 significantly improved hindlimb weight bearing when compared to vehicle. P <0.0001; two-way ANOVA; n =12. **(B)** Hindlimb incapacitance 30 minutes after local administration of URB597 to acutely inflamed knees. The anti-nociceptive effect of URB597 (3.0 mg/kg) was blocked by the CB1 receptor antagonist AM251 (0.2 mg/kg), but not the CB2 receptor antagonist AM630 (0.2 mg/kg). When URB597 was administered to the contralateral knee, there was no observable effect on hindlimb weight bearing. Data are means ± SEM. ^*^ *P* <0.05, ^**^ *P* <0.01; one-way ANOVA with Bonferroni’s *post hoc* test; *n =*9 to 14. ANOVA, analysis of variance; SEM, standard error of the mean.

Based on the results of the inflammation studies, a dose of 3.0 mg/kg was chosen for pain behaviour assessment. URB597 significantly improved inflamed hindlimb weight bearing during the initial 3 hr after administration (*P <*0.05; *n* =12; Figure 5A). The most profound effect of URB597 was observed at 30 min; therefore, subsequent experiments focused on this time point. In mice pretreated with the CB1 receptor antagonist AM251 (0.2 mg/kg; *n =*9), the anti-nociceptive effect of URB597 was abolished (*P* <0.05; Figure 5B). Conversely, pretreatment with the CB2 receptor antagonist AM630 (0.2 mg/kg; *n =*9) had no effect on URB597-induced anti-nociception (*P* >0.05; Figure 5B). In order to confirm that FAAH inhibition was occurring locally in the treated joint and not systemically, URB597 (3.0 mg/kg) was administered to the contralateral joint and, in this instance, the drug had no effect on incapacitance (*P* >0.05; *n =*9; Figure 5B).

When administered alone, neither antagonist had an effect on hindlimb weight bearing when compared to vehicle (*P* >0.05; *n* =6 to 7; data not shown).

### von Frey hair secondary allodynia

**Figure 6:**
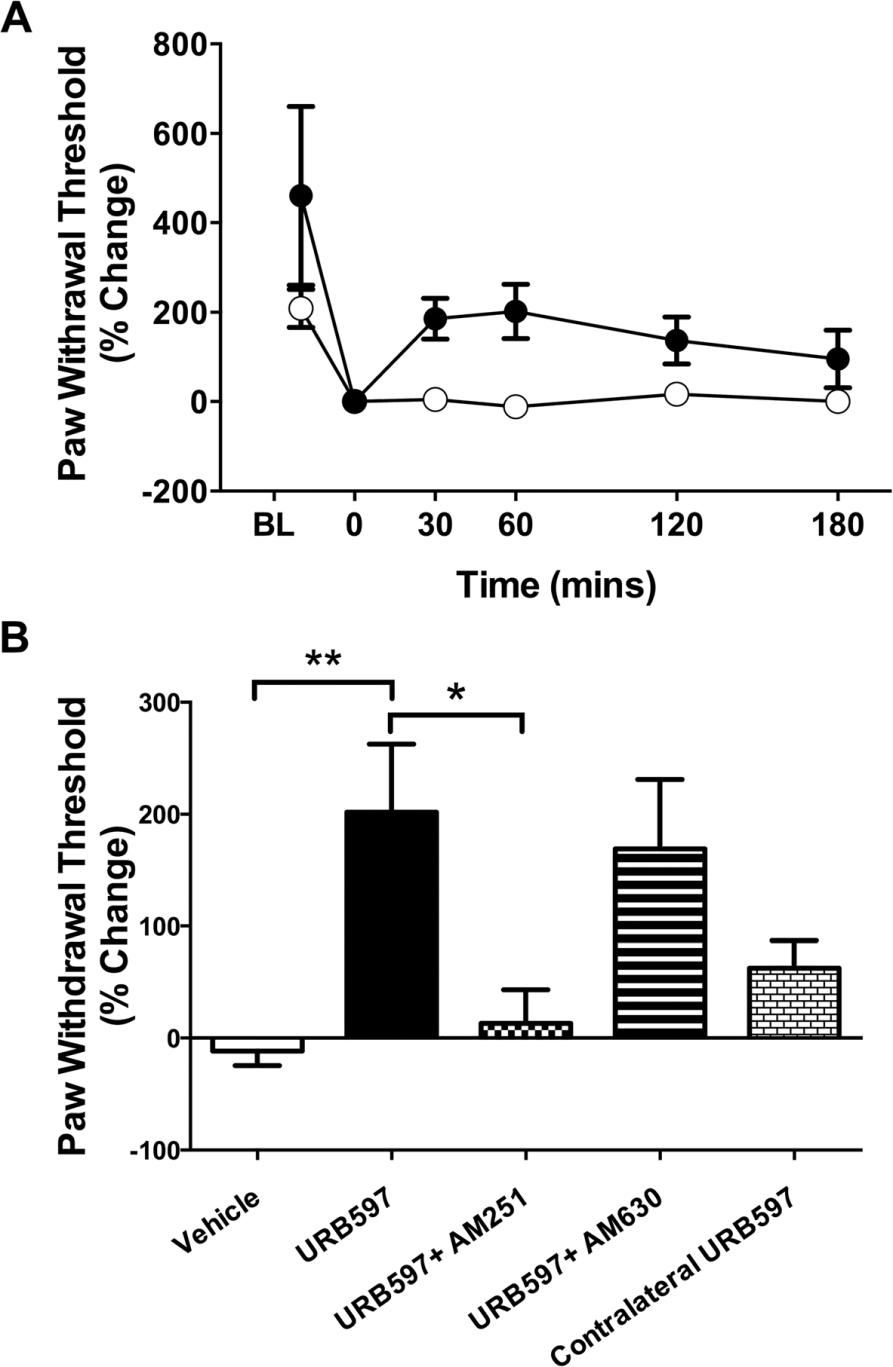
**von Frey hair algesiometry in mice with acute joint inflammation. (A)** Time course of the effects of URB597 (3.0 mg/kg) on inflamed hindlimb von Frey withdrawal threshold. URB597 significantly improved hindlimb withdrawal thresholds when compared to vehicle. *P* <0.0001; two-way ANOVA; *n* =12 to 14. **(B)** Inflammation-induced secondary allodynia 60 minutes after local administration of URB597 (3.0 mg/kg), as tested by von Frey hair algesiometry. URB597 significantly improved mechanosensitivity. This effect was blocked by AM251 (0.2 mg/kg), but not AM630 (0.2 mg/kg). Contralateral administration of URB597 had a negligible effect on ipsilateral tactile sensitivity. Data are means ± SEM. ^*^ *P* <0.05, ^**^ *P* <0.01; one-way ANOVA with Bonferroni’s *post hoc* test; *n =*9 to 14. ANOVA, analysis of variance; SEM, standard error of the mean.

Over the course of 1 hr, URB597 (3.0 mg/kg) significantly increased paw withdrawal thresholds when compared to vehicle (*P <*0.05; two-way ANOVA; *n* =12 to 14; Figure 6A). Paw withdrawal thresholds returned to baseline after 2 to 3 hrs; therefore, further experiments focused on the 1 hr time point. The anti-allodynic effect of URB597 was blocked by pretreatment with the CB1 receptor antagonist AM251 (*P* <0.05; *n* =11; Figure 6B), but not the CB2 receptor antagonist AM630 (*P* >0.05; *n =*13; Figure 6B). When URB597 was administered to the contralateral knee, the anti-allodynic effect of the FAAH inhibitor was negligible (Figure 6B).

## Discussion

The medicinal value of *Cannabis sativa* has been known for thousands of years [28]; however, scientists have only begun to uncover the endogenous mechanisms that underlie these beneficial effects. Based on the results presented here, the endocannabinoid system is capable of decreasing joint hyperaemia, leukocyte trafficking and pain during acute inflammatory events.

### The effects of URB597 on inflammation

Activation of the endocannabinoid system has been shown to decrease the development of inflammation in the intestine [29], central nervous system (CNS) [30] and hindpaw [31]. More specifically, cannabinoids are capable of reducing leukocyte proliferation and cytokine release [32,33]. In our study, inflammation was measured *in vivo* by visualizing the interactions between leukocytes and the vascular endothelium, as well as measuring changes in vascular perfusion in acutely inflamed mouse knee joints. When kaolin/carrageenan-inflamed knee joints were treated with low-dose URB597, there was a significant reduction in the number of rolling and adherent leukocytes, and inflammation-induced hyperaemia. A similar effect has been observed in the cerebral [34,35] and ocular [36] microcirculation, where synthetic cannabinoids decreased leukocyte rolling and adhesion. However, as dosing increased, the effects of URB597 on leukocyte kinetics and hyperaemia were less pronounced. One possible explanation for this effect is the downregulation and/or desensitization of articular cannabinoid receptors as a result of excessive endocannabinoid accumulation in the joint [37]. Alternatively, high doses of URB597 may be having off-target effects, which promote inflammation and pain thereby masking any beneficial responses. For example, high concentrations of anandamide are known to cause peripheral sensitization in joints by opening vanilloid-gated ion channels [38]. Furthermore, anandamide can be oxygenated via a cyclooxygenase-2 pathway leading to the formation of pro-inflammatory and algesic prostamides, which may offset the therapeutic benefits of FAAH inhibition [39,40]. Therefore, URB597 may need to be administered with selective prostamide antagonists in order to achieve greater pain relief and resolution of inflammation.

In the present study, the inhibitory effects of URB597 on leukocyte rolling and blood flow were blocked by both CB1 and CB2 receptor antagonism, while the reduction in leukocyte adhesion was CB receptor independent. The inhibitory effect of URB597 on inflammation could be due to a decrease in joint nerve activity following CB1 receptor activation. It has been shown that CB1 receptors are highly expressed on knee joint nerve terminals [17] and that selective activation of CB1 receptors can cause a decrease in the neuronal release of pro-inflammatory neuropeptides, such as calcitonin gene-related peptide (CGRP) [41]. Since CGRP promotes leukocyte migration [42] and vasodilatation [43], it is possible that the anti-inflammatory effects of URB597 described here are due to a CB1 receptor-dependent reduction in CGRP release although other pro-inflammatory neurogenic factors could also be involved.

The present investigation found that CB2 receptors are involved in endocannabinoid-mediated inhibition of rolling and hyperaemia in inflamed joints. This CB2-mediated inhibitory effect on leukocyte rolling has previously been observed in the retina [44]. The CB2 receptor agonist JWH-133 has been shown to inhibit the expression of P-selectin [44], an adhesion molecule responsible for the initiation and maintenance of leukocyte rolling [3]. It is, therefore, possible that URB597 decreased leukocyte rolling by promoting the inhibitory effect of the endocannabinoid system on selectin expression.

The inability of CB receptor antagonists to block the inhibitory effects of URB597 on leukocyte adhesion is intriguing. While cannabinoids classically bind to either CB1 or CB2 receptors, atypical cannabinoids have been found to bind to alternate G protein-coupled receptors such as GPR55 and GPR18 [45]. Both of these receptors have been found to have immunoregulatory functions [46] and, in the brain, GPR18 stimulation has been shown to inhibit leukocyte recruitment [47]. It is, therefore, possible that URB597 is causing the accumulation of atypical endocannabinoids in the joint whose activity reduces leukocyte adhesion through either or both of these atypical cannabinoid receptor subtypes.

The changes described here, concerning leukocyte adhesion, are in contrast to a study by Ni and colleagues [35], who found that cannabinoid-mediated leukocyte anti-adhesion was CB2 receptor-dependent and the inhibitory effect on leukocyte rolling was CB1 receptor-independent. The reason for the disparity between the two studies could be that Ni *et al.* were investigating leukocyte changes in response to a non-selective CB receptor agonist (WIN5512-2) and activity was assessed in the brain. Thus, there may be subtle differences in cannabinoid receptor properties between the microcirculation of the CNS versus peripheral tissues.

### The anti-nociceptive effects of URB597

The results presented here show that URB597 can decrease inflammatory pain, and are in accordance with several other studies that focused on the role of endocannabinoids in treating pain. The attenuation of hindlimb incapacitance suggests that URB597 is having a direct effect on functional pain recovery in the joint, while the improvement in paw withdrawal threshold is indicative of a reduction in referred pain (secondary allodynia). Systemic FAAH inhibition has been shown to decrease animal pain behaviours in a preclinical model of OA [25]. However, when URB597 was administered locally to the joint, it reduced nociceptor activity, suggesting a peripheral site of action of endocannabinoids [25]. Since contralateral administration of URB597 had no discernible effect on hindlimb incapacitance and anti-allodynia here, it can be inferred that the anti-nociceptive effects of FAAH inhibition are locally mediated. The potential advantage of using URB597 peripherally is that it could allow the accumulation of endocannabinoids in the synovium of affected joints and avoid the potential psychotropic side effects of cannabinoid receptor activation in the CNS.

The analgesic effects of URB597 were blocked by a CB1 antagonist, and this result corroborates other reports of CB1 receptor-mediated analgesia [48]. The analgesic effects of CB1 receptor activation are thought to primarily involve spinal [49] and supraspinal [50] CB1 receptors. Although cannabinoids can reduce pain responses via the activation of CB1 receptors in these higher centres, the data presented in this study would suggest that peripheral CB1 receptors are also able to elicit an anti-nociceptive effect in joints. This hypothesis is supported by the observation that the selective CB1 receptor agonist arachidonyl-2′-chloroethylamide (ACEA) significantly decreased the nociceptor firing rate when applied locally to OA knee joints [19].

While the preclinical evidence supporting the therapeutic potential of the endocannabinoid system is compelling, a recent clinical trial involving OA patients was less encouraging [51]. Treatment of OA subjects with the FAAH inhibitor PF-04457845 failed to improve reported pain scores when compared to placebo. One of the disparities of this study compared to the preclinical findings is that PF-04457845 was given systemically rather than locally into the joint and as such could be imparting off-target effects, particularly in the brain. In addition, the patients used in the trial were from a heterogeneous OA population and were not stratified based on the degree of synovitis, neuropathic involvement, or disease severity. The data presented here indicate that FAAH inhibitors may be more efficacious in OA patients who suffer from acute inflammatory flares and ongoing synovitis.

## Conclusions

In summary, the potent and selective FAAH inhibitor URB597 was shown to have both anti-nociceptive and anti-inflammatory effects in the kaolin/carrageenan model of joint acute inflammation. These effects were local and appear to be mediated by distinct cannabinoid receptor mechanisms. Since FAAH breaks down several fatty acid amides including anandamide, OAE and PEA, future work is required to identify the specific endocannabinoids responsible for reducing acute synovitis and pain. Nevertheless, pharmacological promotion of endocannabinoid activity could be a useful way to treat inflammatory joint flares.

## Abbreviations

ACEA: arachidonyl-2′-chloroethylamide
ANOVA: analysis of variance
CAM: cell adhesion molecule
CB: cannabinoid
CGRP: calcitonin gene-related peptide
CNS: central nervous system
FAAH: fatty acid amide hydrolase
IVM: intravital microscopy
LASCA: laser speckle contrast analysis
OA: osteoarthritis
OAE: N-oleoylethanolamine
PEA: palmitoylethanolamine
RA: rheumatoid arthritis
SEM: standard error of the mean
